# Experimental Study of Flame Extinguishing Using a Smart High-Power Acoustic Extinguisher: A Case of Distorted Waveforms

**DOI:** 10.3390/s26041204

**Published:** 2026-02-12

**Authors:** Jacek Lukasz Wilk-Jakubowski

**Affiliations:** Department of Information Systems, Kielce University of Technology, 7 Tysiąclecia Państwa Polskiego Ave., 25-314 Kielce, Poland; jwilk@tu.kielce.pl

**Keywords:** environmentally friendly flame extinguishing, high-power acoustic extinguisher, low-frequency waves, sensors, intelligent module, fire safety, zero-waste technology

## Abstract

The acoustic technique emerges as a highly promising, cutting-edge solution that can be effectively employed for extinguishing flames in locations where the access to classical fire-protection measures is limited, the available extinguishing agent is severely restricted, or the burning materials are difficult to suppress using currently known methods. The results of the experimental attempts confirmed that low-frequency acoustic waves containing higher even harmonics from the tenth to the sixteenth order (inclusive) can successfully extinguish flames, demonstrating both the feasibility and the novelty of the acoustic technique for fire protection. Moreover, statistical analysis was applied to identify operational boundary values and assess their variability, supporting the optimal selection of system parameters required for rapid and effective flame extinguishing. By integrating an acoustic extinguisher with optional intelligent sensors, including artificial vision, it becomes possible to rapidly detect flames at much greater distances than with conventional smoke and temperature sensors, as well as to automatically extinguish them. In this context, an integrated solution combining acoustic waves with an artificial intelligence module (smart sensor) may be employed for comprehensive fire management, encompassing both fire detection and flame extinguishing.

## 1. Introduction

Because the losses caused by fires are substantial not only in economic terms, there has been a continuous development of innovative techniques for flame detection and fire suppression in recent years. As a result, the issues of early fire detection and effective response have attracted the attention of many researchers worldwide. Modern fire safety systems increasingly incorporate the latest advances in science and technology, including advanced sensor systems, Internet of Things (IoT)-based environmental monitoring, and deep learning algorithms. These solutions enable rapid fire detection and, consequently, faster and more effective fire suppression [1,2,3,4,5,6,7].

In practice, fire detection and extinguishing are particularly critical in hard-to-reach locations. Therefore, increasingly innovative and sophisticated methods for optimizing fire safety systems, as well as flame detection and extinguishing technologies, are being developed to enhance safety, including in buildings [8,9,10,11]. Significant research efforts are also focused on the use of acoustic flame-suppression technologies as a contactless, portable, and environmentally friendly method of firefighting [10,11]. Unlike conventional firefighting approaches, such systems can operate continuously and do not leave behind difficult-to-remove residues, making them a potential alternative to traditional fire extinguishers that rely on standard extinguishing agents—especially in confined or inaccessible spaces or in situations where the amount of extinguishing material is severely limited. Furthermore, there is a growing adoption of solutions that support emergency services through automation and intelligent assistance in hazardous environments, with applications in fields such as crisis management [12,13]. In practice, in addition to fires occurring in enclosed spaces, fires also arise in open environments, including forested areas. The problem of fire occurrence is so significant that in 2022 alone, the reported fire damage covered 1.6 million acres in Europe, an area equivalent to one-fifth of Belgium [14]. Fires usually occur as a result of plants drying out, as the forest floor becomes dry in autumn and winter. In recent years, numerous fires have appeared in areas where they had not been previously reported on such a large scale (including Germany, Romania, and Austria) [14]. They can be caused by many factors, including human activity (even field work carried out near forest areas). Therefore, it is not surprising that innovative and environmentally friendly ways to combat fires are being sought worldwide [15,16,17].

As mentioned earlier, one of these approaches is the acoustic method, which appears to be an emerging technique that, if not at present, may in the future have the potential to replace or support traditional flame extinguishing processes [18,19,20,21,22,23,24,25,26,27,28,29]. Research into its use has been the focus of DARPA’s efforts, which, in 2008, after the fire on the aircraft carrier USS George Washington that caused a considerable financial loss of about $70 million, launched a program focused on the search for fast and effective methods of firefighting [18]. Unfortunately, such incidents remain frequent, and fires in ship holds or aircraft cockpits pose a serious threat to human life and health while causing substantial material losses. This underscores the necessity and growing scientific interest in research aimed at developing innovative firefighting solutions. In practice, as shown in the “Mythbusters” program, it becomes possible to extinguish the flames even with an amplified modulated voice. However, the achieved sound pressure levels exceed the pain threshold of the human ear [30]. Therefore, numerous research projects are currently being conducted to develop sophisticated firefighting techniques at many scientific and academic centers worldwide. Such research is conducted especially through international collaboration with scientists from Poland, Bulgaria, Spain, Ukraine, and Turkey.

The main contribution of this work is to present findings on the potential use of distorted low-frequency acoustic waves containing higher even harmonics for flame suppression, which constitutes a scientific novelty by demonstrating the effective use of distorted waveforms for flame extinguishing (contributing to the state-of-the-art). This is the core Materials and Methods Section (Section 2). The primary novelty of this work lies in the experimental investigation of whether low-frequency acoustic waves, characterized by higher even harmonics from the tenth to the sixteenth order (inclusive) and driven by high-to-very-high power levels, can successfully extinguish flames. The conducted experimental results confirm that these distorted low-frequency waves can effectively suppress flames, demonstrating the feasibility and novelty of this approach for fire management.

In this context, the relationship between frequency and sound pressure level for a low-frequency sinusoidal waveform—contingent upon the presence of even harmonics—is presented. This analysis allows for the estimation of the required pressure based on the parameters of the quenching wave, thereby addressing a significant gap in the existing literature. The values determined through the experimental means may serve as a reference for flame suppression in real-world fire scenarios, while emphasizing the importance of the specific sound pressure level at the point of extinction. The experimental results obtained from the flame-extinguishing attempts using distorted acoustic waves are compared, and the potential applications and benefits arising from the use of this technology are outlined.

Additionally, the paper presents a combination of a high-power acoustic fire extinguisher with a modern deep neural network-based approach. In practice, research on the sensors and fire-extinguishing systems has been ongoing since 2022. However, it should be noted that the detection of flames using computer vision is not a novel concept; numerous studies exist on this subject, several of which are included in the bibliography of this article. The described intelligent sensor module represents an additional optional component of the acoustic extinguisher, whose effectiveness is directly linked to the continuously evolving architecture of artificial vision systems and the selection of appropriate deep learning algorithms. For this reason, the article includes only the most essential general information. In practice, the smart module utilizing deep neural networks can be applied to monitor the environment and detect changes in temperature, smoke, or other indicators of potential fire. Since these solutions are still under evaluation, ongoing work aims to improve performance and reliability metrics, enhancing flame detection capabilities.

### 1.1. Capabilities of Acoustic Wave-Based Flame Extinguishing

**Figure 1:**
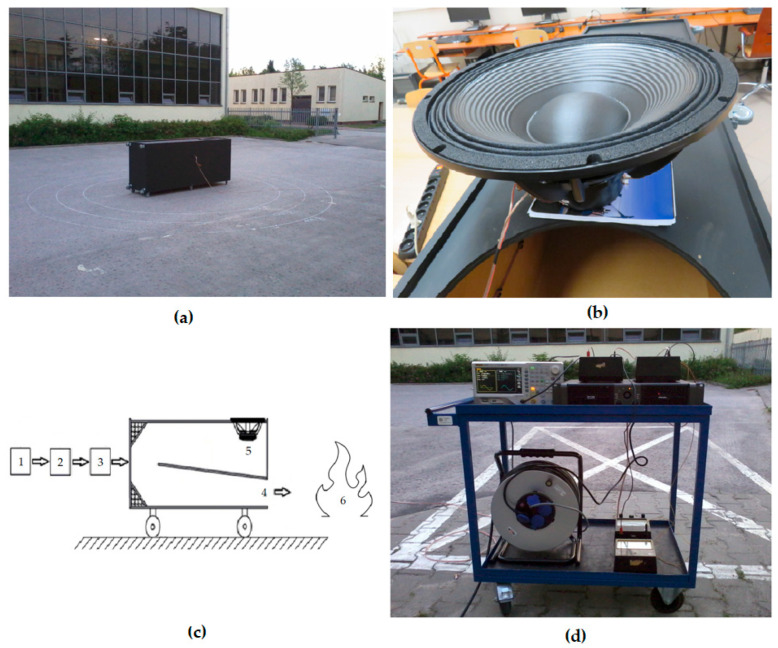
(**a**) Fire extinguisher (photograph of the actual device); (**b**) high- and very-high-power sound source (photograph of the actual device); (**c**) components of the fire extinguisher: (1) wave generator, (2) modulator, (3) amplifier, (4) extinguisher tip (output), (5) sound source (beginning of the waveguide), and (6) source of flames; and (**d**) laboratory station with instrumentation. * As the wave generator and modulator, a Rigol DG4102 (Rigol Technologies, Warsaw, Poland) was used; the power amplifier was a Proel HPX2800 (Proel s.p.a., Sant’Omero, Italy); and the sound source was a B&C 21DS115 loudspeaker (B&C Speakers s.p.a., Bagno a Ripoli, Italy). ** It should be noted that the loudspeaker does not operate as a direct sound source, but as an element of a resonant system. This means that the transfer characteristic of the driver alone does not directly determine the final result, since the final outcome is the combined effect of two elements: the enclosure and the loudspeaker, which together make it possible to achieve the desired frequency range. In this context, the enclosure that was built represents the optimal solution, as it provides high efficiency for infrasonic frequencies.

Figure 1 shows a high- and very-high-power acoustic fire extinguisher together with its associated instrumentation, including a mobile laboratory station. In practice, this research area is relatively new, and experiments are still ongoing to achieve a deeper understanding of the flame-suppression capabilities of acoustic waves. This understanding is crucial for the development of acoustic fire extinguishers, as well as for studying the fundamental principles and natural mechanisms of acoustic wave propagation. In fact, modifying the parameters of the extinguisher—including the characteristics of the generated acoustic wave—directly translates into its extinguishing performance, including the ability to suppress flames at significantly greater distances. In practice, the acoustic extinguisher is designed to generate low- and very-low-frequency waves, including infrasound, as these waves demonstrate advantageous properties for flame suppression. In the case of laboratory tests, very good results were achieved for frequencies below 20 Hz, which is due to the fact that the greatest turbulences and disturbances of the flame front are observed for low frequencies.

The extinguisher is universal—it has frequency-adjustable abilities, a modulating function, so that the parameters of the extinguishing wave can be adjusted depending on the source of the flames. Because acoustic waves penetrate solids, liquids, and gases, appropriately generated and modulated waves of high and very high power can be effectively used to extinguish flames from different materials. An undoubted benefit of using a high-power and very high-power sound source is the ability to increase the distance between the flame source and the extinguisher outlet [16,17,19,20,23,24,25,31]. When the principle of operation is analyzed, the fire extinguisher generally uses the vibration of the diaphragm of a sound source caused by a coil and a magnet. This leads to a disruption of the convection process as the speaker’s diaphragm sets air molecules in motion at the appropriate frequency, which leads to the generation of waves. Namely, the tilt of the diaphragm of the sound source from its equilibrium position imparts motion to the air molecules (the pressure level increases locally), and the molecules transfer their energy to neighboring molecules, also setting them in motion. From a theoretical point of view, the pressure in front of and behind the diaphragm has the same absolute value, but it is phase-shifted by 180 degrees. When the diaphragm of the sound source reaches full tilt, its movement occurs in the opposite direction (the pressure level in front of the diaphragm decreases locally), resulting in the turning back of the particles (negative pressure). The purpose of the loudspeaker enclosure is therefore to isolate the pressure generated by the rear side of the diaphragm from that at the front, so as to prevent the cancellation of waves (i.e., acoustic short-circuiting). In practice, the finding of regularities that occur may depend on the source of the flames [25], which justifies continuing this type of research in the future.

**Figure 2:**
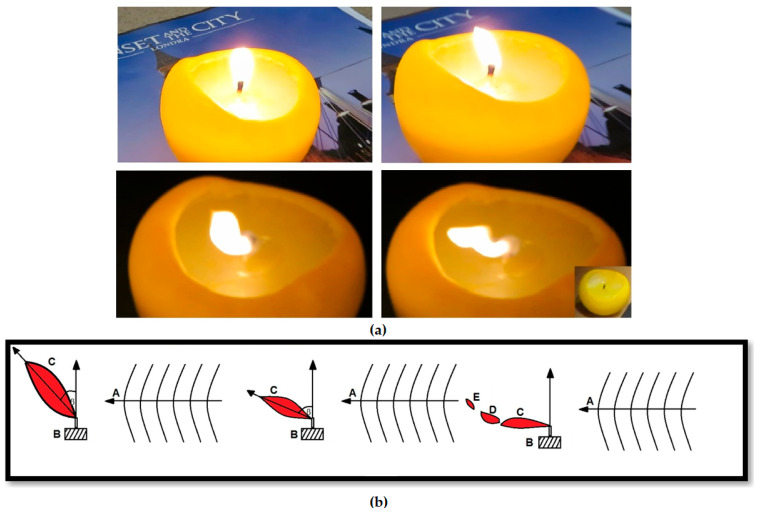
(**a**) Illustration of flame extinguishing using acoustic waves; (**b**) schematic visualization illustrating the physical phenomenon of flame extinguishing by acoustic waves. * (**a**) shows, at the top, the undisturbed flame (upper left corner) and the flame exposed to acoustic waves at a frequency different from the critical frequency (upper right corner). At the bottom, an illustration of the extinction of a disturbed flame subjected to acoustic waves with a frequency close to the critical frequency (lower left corner) and with a frequency equal to the critical frequency (lower right corner) is presented (to improve readability, the lower images are shown against a black background). An image of an extinguished flame is included in the lower right corner. ** In (**b**), letter A denotes the direction of wave propagation; letter B indicates the flame source; C represents the flame deflected from its original plane (equilibrium position) as a result of exposure to acoustic waves (flattening parallel to the direction A of acoustic wave propagation); D denotes the flame rift; and E denotes the exhaust fumes.

The operation of the extinguisher is based on the gradual deflection of the flame (for a certain frequency) and then breaking it into pieces. In practice, a number of substances are noted in the air, such as O_2_, CO_2_, NOx (mainly nitrogen oxide and dioxide), and smoke particles, which affect the extinguishing process. The significance includes both the disruption of airflow due to acoustic emission (this phenomenon can be used to control, including weakening and extinguishing flames, since air oscillations interfere with the supply of oxygen, which is necessary for the occurrence of fire according to the combustion triangle), the dispersion of heat over a larger area, thereby reducing the flame temperature below the critical point, and disrupting the delivery of fuel to the flame. The flame follows the flow of fuel, and at the critical pressure level due to the emission of acoustic waves, it is extinguished (first it is deflected, distorted, dispersed, and then torn, that is, divided into parts and extinguished) [23,24,31,32]. In turn, the removal of heat from the flame (i.e., different dynamics) results in a reduction in temperature. When the temperature of the stripped portion of the flame falls below the ignition temperature, the flame is extinguished. Increased fuel evaporation gives rise to flame expansion and a decrease in its overall temperature. A detached or deflected flame is easier to extinguish than an undisturbed one. In practice, the exhaust fumes are isolated from the combustible mixture, and the flame is eventually extinguished once the mixture is separated from it. For illustrative purposes, the flame extinguishing process is shown in Figure 2.

To date, the physical characteristics and propagation mechanisms of acoustic waves have been analyzed, including the formation of standing waves, interference effects, and phenomena accompanying wave propagation in both confined and unconfined spaces. In addition, the influence of selected parameters and natural phenomena on flame extinguishing has been investigated, the state-of-the-art in acoustic flame extinguishing has been reviewed, and existing technologies and solutions have been analyzed. Scientific efforts have focused not only on the consideration of physical quantities and the analysis of waves with specific parameters in terms of their extinguishing ability, but also on the description of the operation of the extinguisher and the study of the processes occurring during extinguishing. On the basis of the developed structural design and concept of a non-invasive acoustic extinguisher using natural mechanisms of sound wave propagation to extinguish flames, and as part of pre-implementation activities, an extinguisher was built and tested. In addition, attention was paid to the components and construction materials for the extinguisher, as well as to the benefits of the acoustic method. In the long run, this will allow one to detail the phenomena that accompany the propagation of acoustic waves.

### 1.2. Acoustic Flame Suppression Enhanced by Smart Sensor and an Artificial Vision System

**Figure 3:**
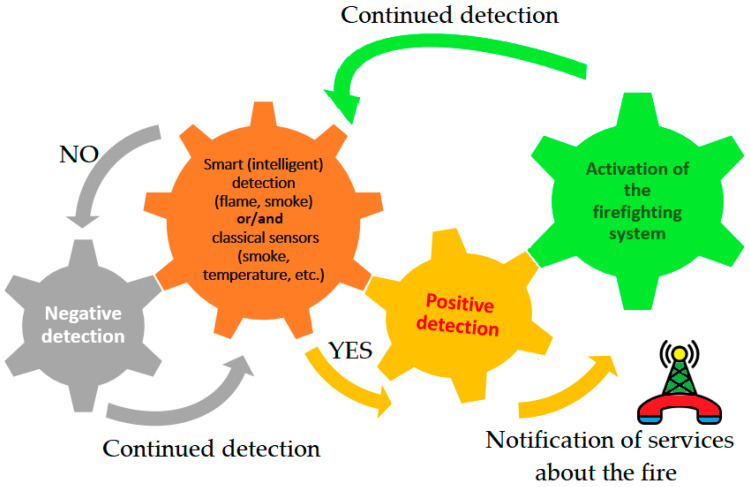
The principle of the fire management system, which includes automatic detection and extinguishing of flames.

Undoubtedly, there is the benefit of being able to use artificial intelligence by attaching an optional fire-detection module to the extinguisher. Such systems can integrate an acoustic extinguisher, an intelligent computer module, and a data processing unit, e.g., CPU (Central Processing Unit), VPU (Versatile Processing Unit), and GPU (Graphics Processing Unit). The key issue is the rapid detection of flames. In practice, fire observation points may be located within buildings as well as in forested areas, such as elevated observation towers used to monitor smoke and fire. From the perspective of firefighting operations, the shorter the response time, the more effective the suppression effort. International research efforts are focused on developing an intelligent acoustic fire extinguisher that aims to monitor and analyze data from various outlets, such as visible and infrared sensors [33]. Such a system may identify fire or smoke based on anomalies in the color of pixels, and in the event of a positive fire or smoke detection, immediately notify the fire protection services. The benefit then is that humans do not have to monitor the environment themselves or even be present during ongoing firefighting operations. This becomes possible thanks to visual detection, which allows flames to be detected at much greater distances than when using typical smoke and temperature sensors [34,35,36,37,38,39,40,41,42,43]. Furthermore, in the case of positive detection, relevant services can be immediately informed, and the artificial intelligence-supported firefighting system can be automatically activated [33,35,36,42,44,45]. In this sense, the acoustic technique can be applied not only to extinguish flames, but also to detect them (using intelligent sensors) [23,24,25,31,32,33,35,36,42,44,45]. The principle of a fire protection system that includes both aspects of fire management, that is, automatic detection and flame extinguishing, is provided in Figure 3. In practice, such a system can run continuously until the flames are extinguished.

**Figure 4:**
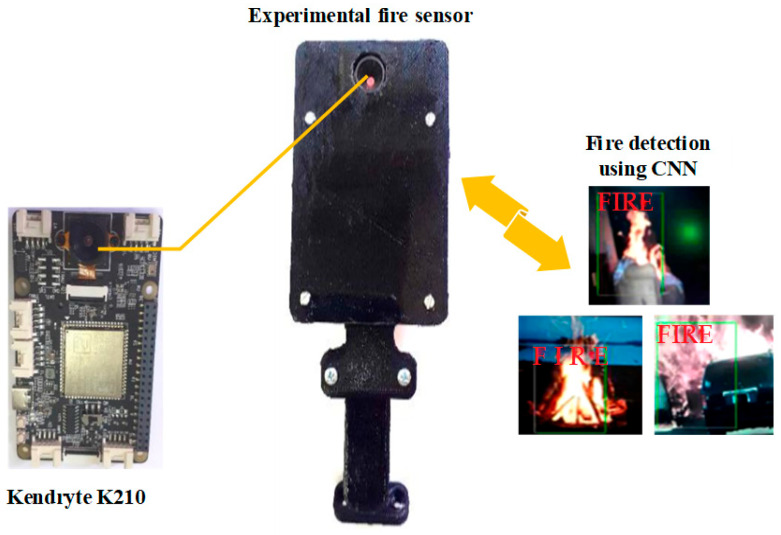
Experimental sensor for flame detection.

A smart sensor for flame detection, developed in a scientific collaboration at the Technical University of Gabrovo and intended for use in acoustic-based extinguishing systems, is shown in Figure 4. In practice, the processor incorporates a neural network capable of detecting flames [33].

An interesting research direction is information processing in open space, including monitoring, early observation, and acoustic characteristics of many factors [46,47,48,49,50,51,52,53,54,55,56,57,58]. It is important to take into account specific propagation conditions, depending on the frequency band used for communication and the method of data transmission [59,60,61,62,63,64,65,66,67,68,69,70,71,72,73,74]. As shown in [75], based on the characteristics of the flames, it is possible to develop a decision support system using machine learning, which can ensure fast and effective flame extinguishing.

In practice, apart from the works cited in the References Section, there are few experimental studies devoted to the use of acoustic waves generated by high- and very-high-power sound sources. A separate, but equally important group of studies is those on the analysis of combustion processes [76,77,78,79,80,81,82,83,84,85,86,87,88]. For this reason, there is a clear need to address the existing theoretical and practical gap in the literature in this area.

From a practical point of view, the acoustic technique for extinguishing flames uses both modulated and unmodulated waves with different shapes and frequencies [16,17,19,20,23,24,25,31,32,35]. Moreover, in the acoustic method, signals with changing frequencies can be applied to extinguish flames from different materials. The acoustic method allows the extinguishing of fires of different classes [19,23,25,89]. While some studies analyze the use of acoustic waves for flame extinguishing at short distances, the tested fire extinguisher enables a significantly greater extinguishing range. In this study, experiments were conducted at a distance of 50 cm. This is important for understanding the extinguishing capabilities of low-frequency acoustic waves. Many researchers have highlighted the need for large-scale investigations, indicating a clear gap in the existing literature that this study seeks to address [76,90,91].

The field of the project under development [92], co-financed by the Polish Ministry of Science and Higher Education of the “Innovation Incubator +” (Inkubator Innowacyjności +) program, is primarily acoustics and electronics. The research and development work underway involves testing of the acoustic extinguisher (taking into account the conceptual apparatus of acoustics), which, in the long run, can contribute to a better understanding of the extinguishing capabilities of low-frequency acoustic waves generated from a high-power sound source, as well as within the framework of international cooperation to develop an intelligent module (using artificial intelligence). The key benefit is the ability to rapidly detect flames and initiate firefighting actions without unnecessary delay. Therefore, the ongoing research conducted within the project has significant practical potential. Complementing this project are studies carried out in collaboration with researchers from Bulgaria, aimed at developing an intelligent sensor that can be integrated into the fire extinguisher as an additional module, enabling flame detection and the automatic activation of acoustic flame suppression upon detection [17,33,35,36,42,44,45].

To summarize, the operating principle of the acoustic fire extinguisher has been explained, while the project is currently undergoing thorough testing. This technique can be applied as a complementary firefighting method or as a means of controlling flames and suppressing the combustion process, including the creation of buffer zones. In practice, a combination of multiple firefighting techniques can be used, such as acoustic waves and water mist [93]. The acoustic fire extinguisher may have additional (optional) elements that support constructive interference (such as baffles, barriers, wave-reflecting elements, pressure-boosting caps, transducers, and energy banks, etc.) [25,87,94]. It is also possible to connect several extinguishers together, thus creating an acoustic system. Additionally, an extinguishing system that uses acoustic waves can be connected to an acoustic warning system, which includes classical smoke and temperature sensors. Based on the obtained data, depending on the parameters of the generated acoustic signal (including frequency and waveform, type of modulation, and the order of harmonics), it is possible to measure the sound pressure value at which the flames were successfully extinguished, as well as the power that must be delivered to the sound source to extinguish the flames. The study reported in [89], in which attempts were made to generate even harmonics, highlighted the need for continued research on higher-order harmonics. In practice, the technical parameters of the extinguishing acoustic wave, including the operating frequency and harmonic order, can be adjusted. This enables an analysis of the flame-extinguishing capacity of acoustic waves using distorted waveforms. This makes it possible to address the question of whether flame extinguishing is achievable using such distorted acoustic waves and how the harmonic order influences the extinguishing capabilities of low-frequency acoustic waves.

This article includes experimental results that cover higher even harmonics from to the tenth up to the sixteenth order (inclusive), which fills the existing gap in this area and may contribute to a better understanding of the extinguishing capacity of low-frequency acoustic waves. It is worth noting that in this article, low-frequency acoustic waves with higher tenth- to sixteenth-order even harmonics are understood to be distorted waveforms containing, in addition to the fundamental component, higher-even harmonics up to the tenth and sixteenth orders (inclusive), respectively.

The study includes diagrams showing sound pressure values observed at the point of flame extinguishing, as a function of frequency and order of higher harmonics, minimum electrical power values that had to be delivered to the extinguisher to extinguish the flames, fast Fourier transform (FFT) graphs, histograms showing the frequency distribution for equivalent sound level values, equivalent sound level values using Z-correction characteristics, and boundary levels exceeded by the temporary noise level during no more than n% of the observation period.

## 2. Materials and Methods

For the purposes of the study, several research methods were applied, which were selected for their use to achieve specific research goals. In the conceptual approach, elements of system analysis were applied, reflecting the research objective of examining the influence of specific factors—such as frequency and harmonic order—on flame extinguishing. The system analysis allowed for a certain structure of the problem to emerge, which in turn was reflected in the experiments carried out. In the introduction to the article, the literature review method was applied. In turn, in the practical part, the statistical method was employed, as well as the method of individual cases, which were applied to interpret the results of the firefighting attempts carried out. In addition, the experimental method, data analysis, and the comparative method were used. It should be noted that all the results obtained were compared, which is particularly important from the point of view of evaluating the effectiveness of extinguishing tests using signals with different frequency components. The article also uses elements of the legal institutional method.

In practice, nominal power amplifier parameters are given according to the AES (Audio Engineering Society) requirements. All measurements conducted were compliant with ISO 2631-1 and ISO 2631-2 standards. Acoustic measurements were performed according to the IEC (International Electrotechnical Commission) standards with Class 1 accuracy (IEC 651 and IEC 61672).

### 2.1. Procedures and Conditions of Experimental Attempts

For acoustic measurements, an SVAN 979 (SVANTEK Ltd., Warsaw, Poland) Class 1 m was applied. The Z-correction characteristics and a parallel detector with a time constant of 1000 ms were used. Consequently, an image of the acoustic energy distribution for the analyzed frequencies was obtained. Hanning windowing and linear FFT averaging were applied in the analyses. Interpretation of the obtained results confirmed the presence of harmonic components that affect the extinguishing of flames using properly generated acoustic waves. In practice, as demonstrated in this article, it is possible to extract individual components of the acoustic signal, which is important for analyzing their influence on flame extinguishing. For safety reasons, the power amplifier used was equipped with thermal protection to safeguard the device against hazardous operating conditions. The acoustic extinguisher employed a very high-power sound source, with a nominal speaker power of 1700 W. When waveforms containing even harmonics up to the sixteenth order (inclusive) were tested, thermal protection was tripped at 650 W. In practice, the flames were extinguished using significantly less power than the nominal power of the sound source installed in the extinguisher.

The applied extinguisher uses a rectangular cross-section (see Figure 1). An acoustic wave generator, a power amplifier, and a dedicated waveguide of the appropriate length were applied to emit acoustic waves at the appropriate frequency. The author is aware that better sound transmission conditions could be achieved with a straight waveguide; however, this was one of the design assumptions—the folding of the waveguide was intended solely to reduce the overall dimensions of the enclosure. During the design phase, a dedicated enclosure was developed to achieve high efficiency and realize the intended operating frequency range. In the case of a one-sided closed waveguide, the required waveguide length is twice as short. Therefore, due to practical considerations (reduction in dimensions), it was decided to use a one-sided closed waveguide. All attempts were carried out using the acoustic extinguisher shown in Figure 1, with a waveguide length of 4.28 m (it was selected for an operating frequency of approximately 17.25 Hz). This value corresponds to one quarter of the wavelength of the acoustic wave propagating within the waveguide. The results are for the fundamental frequency changed from 14 to 18 Hz (with a step of 1 Hz) for sine waves. These frequencies were chosen in a non-accidental way—they oscillate around the frequency of 17 Hz for which the extinguisher was designed [20]. All waveforms containing harmonics were generated using a generator. The signal was provided to an amplifier. In practice, other ways to generate the appropriate waveform, such as using an H-bridge, are also possible.

To unequivocally determine whether the flames were successfully extinguished, a point source of flames (paraffin wax candles with a flame height of approximately 2 cm), placed at a distance of 50 cm from the extinguisher outlet, was applied. Within the conducted experiments, the flame source was, in each case, positioned downstream of the outlet of the acoustic extinguisher, as shown in Figure 1. The appropriate parameters of the flame-extinguishing wave were then set, including the fundamental frequency and the harmonic order, after which the measuring instruments and the acoustic extinguisher were activated. First, the fundamental frequency was set, followed by the harmonic series, after which the acoustic extinguisher was activated. The measurement time was 5 s, meaning the sound pressure level was integrated over a 5 s period. For each harmonic order, depending on the frequency analyzed (f parameter), the sound pressure level (SPL) at which the flame extinguishment was recorded, as well as the power value (P) that had to be delivered to the extinguisher to extinguish the flames, were measured. Many measurements of instantaneous peak values, minimum, maximum, and instantaneous rms (root mean square) values were made, and the instantaneous rms values were included in this article. In practice, short-term changes in weather conditions can affect measurement results, as can the location and conditions surrounding the measurements. Therefore, all experiments were performed in an open space, under windless conditions, on the same day. The instantaneous SPL of the background noise associated with the measurement was equal to 59.1 dB.

**Figure 5:**
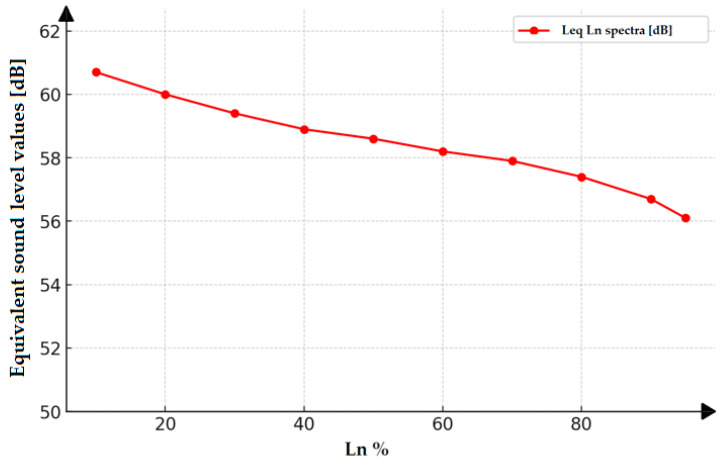
Equivalent sound level values Leq–Ln spectra [dB] and boundary levels (Ln%) exceeded by the temporary noise level for no more than n% of the observation period.

In practice, statistics play an important role in research by providing tools to analyze, interpret, and present data, as exemplified by the use of statistical methods to assess the acoustic conditions at the site of acoustic extinguisher experiments. By using the statistical apparatus, it was possible to determine by how much time the SPL exceeded a certain value. The statistical level Ln% represents the boundary (threshold) level that the background noise exceeded for a maximum of n% of the observation period (Figure 5). Since the pressure level is a random variable, it can be determined with some probability that its instantaneous value will be within a certain range.

The use of statistical values (in this case, equivalent sound level values and boundary levels) makes it possible to determine the acoustic characteristics at different times during the experimental attempts. Based on an analysis of the Leq–Ln spectra [dB] values obtained for specific boundary levels (Ln%), it can be seen that during the measurements of 95% of the observation period, the recorded background noise level was greater than 56.1 dB. In turn, the 60 dB background noise level was exceeded by no more than 20% of the observation period, which means that for 20% of the observation time, the background noise level was greater than 60 dB. The maximum difference was equal to 4.6 dB, indicating that the observed changes in noise levels were small (56.1 dB for L95 and 60.7 dB for L10).

### 2.2. Extinguishing Waveforms Using Distorted Signals

**Figure 6:**
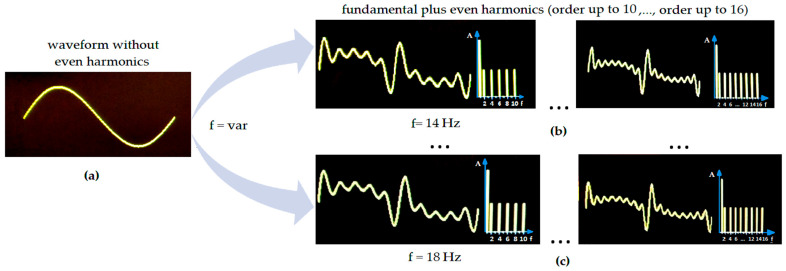
(**a**) Fundamental waveform without even harmonics (f = var = 14…18 Hz); (**b**) fundamental waveform plus even harmonics for the smallest analyzed frequency, i.e., 14 Hz; (**c**) fundamental waveform plus even harmonics for the largest analyzed frequency, i.e., 18 Hz. * The order of even harmonics to which the results were analyzed was changed from up to 10 to up to 16.

Although waves with modified signal components whose frequencies are integer multiples of the fundamental frequency exhibit flame-extinguishing capabilities, this paper presents time- and frequency-domain graphs of flame-extinguishing waveforms that include even harmonics up to the tenth and sixteenth orders (hereafter referred to as boundary waveforms), as shown in Figure 6. The study extends the state-of-the-art in distorted waveforms by presenting experimental investigations of even harmonics and by providing a comparative analysis of the obtained results. This is particularly evident in the section presenting the FFT analyses.

As mentioned earlier, the results for the frequency *f*_1_ changed from 14 to 18 Hz (with a step of 1 Hz) for sine waves. The desired mechanical load was then obtained in the speaker diaphragm, which was an effective use of its power capabilities [89]. Sometimes during measurements, it was observed that for low frequencies, successful extinguishing attempts were not always possible. The reason for that was the significant amplitude of oscillations due to the incompatibility of the waveguide with the sound source (overexcursion of the speaker is an undesirable phenomenon).

The use of the Fourier transform allowed one to transform a continuous function from the time domain to another continuous function in the frequency domain. Using frequency superposition, the Fourier transform was obtained:

(1) $$f(t)=A_{1}sin\left(2\pi f_{1}t+\varphi_{1}\right)+A_{2}sin\left(2\pi f_{2}t+\varphi_{2}\right)+A_{3}sin\left(2\pi f_{3}t+\varphi_{3}\right)+\ldots$$

where *A*_1_ is the amplitude [V] (pick-to-pick), *f*_1_ is the fundamental wave frequency [Hz] (first harmonic, main oscillations), and *φ*_1_ [°] is the initial phase. As Equation (1) shows, the resulting waveform *f*(*t*) is a superposition of a series of sinusoidal waveforms. From a practical point of view, the frequencies of the other harmonics are integer multiples of the frequency *f*_1_ (fundamental oscillations), that is, for even multiples they will be: 2*f*_1_ (second harmonic), 4*f*_1_ (fourth harmonic), 6*f*_1_ (sixth harmonic), 8*f*_1_ (eighth harmonic), 10*f*_1_ (tenth harmonic), 12*f*_1_ (twelfth harmonic), 14*f*_1_ (fourteenth harmonic), and 16*f*_1_ (sixteenth harmonic). The total is the first eight even harmonics. The experimental part presents results from extinguishing attempts using distorted waveforms containing even harmonics of higher orders (i.e., up to and including the sixteenth order). As demonstrated, the generated extinguishing signals that effectively suppress flames may contain a fundamental component and, in addition, harmonic components (in the case of even harmonics up to the tenth order, these are the following multiples of the *f*_1_ frequency: 2*f*_1_, 4*f*_1_, 6*f*_1_, 8*f*_1_, and 10*f*_1_; in turn, in the case of even harmonics up to the sixteenth order: 2*f*_1_, 4*f*1, 6*f*_1_, 8*f*_1_,10*f*_1_, 12*f*_1_, 14*f*_1_, and 16*f*_1_). Graphs of flame-extinguishing waveforms in the time and frequency domains, plus even harmonics up to the tenth and sixteenth orders, are presented in Figure 6.

It should be noted that the power delivered to the extinguisher is the power provided to the sound source, which is an essential component of the acoustic extinguisher. To estimate (predict) the power P that must be supplied to the extinguisher in order to achieve complete flame extinguishment, as well as the SPL value at the location where extinguishment is observed, empirical formulas can be applied using statistical methods, taking into account the nonlinear nature of the phenomena involved. In turn, the analysis of the results obtained for different fundamental frequencies and harmonic orders makes it possible to determine the most favorable extinguishing conditions.

The aim of the experiments in the next section is to demonstrate whether it is possible to extinguish flames using distorted acoustic waves, which, in addition to the fundamental frequency, contain harmonic components of higher orders (up to and including the sixteenth order). On this basis, it will be possible to determine the effect of harmonic order modification on flame extinguishing. All experimental results presented in this article are derived from real extinguishing attempts and are not based on simulations.

## 3. Results and Discussion

By employing acoustic waves whose parameters (e.g., frequency, modulation, and harmonic order) can be adjusted according to the flame source, effective flame extinguishing can be achieved. In this study, the sequence of the orders of higher-even harmonics contained in the signal was changed from two to sixteen, with harmonics of the tenth and sixteenth orders being understood as boundary harmonics. This means that the resulting waveforms, in addition to the fundamental frequency, contain higher harmonics up to the tenth and sixteenth orders (inclusive), respectively, and therefore, the results presented in Table 1, Table 2, Table 3 and Table 4 refer to distorted waveforms, including even harmonics up to the tenth and sixteenth orders. They present results in the form of SPL values as well as the P values required to extinguish flames as a function of frequency (f) and even harmonics (up to the 10th and 16th orders, respectively) for a low-frequency sinusoidal waveform.

Table 1 shows the levels of sound pressure measured at certain frequencies at which the flame extinguishing attempts were successful. In turn, Table 2 contains the power values that had to be delivered to the extinguisher to completely extinguish the flames. This conceptual approach made it possible to, in the long term, see some regularities in the sound pressure and power that had to be delivered to the acoustic extinguisher to completely extinguish the flames.

**Figure 7:**
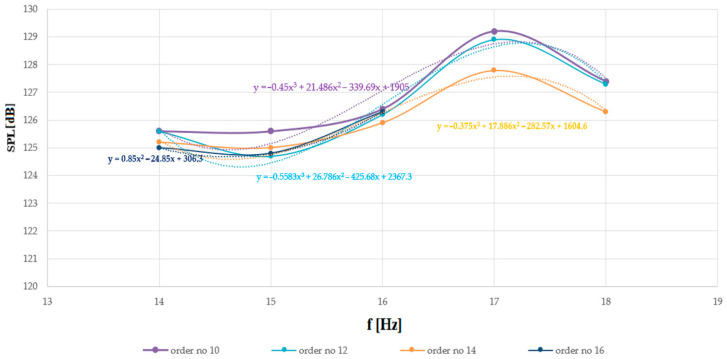
SPL [dB] versus f [Hz] depending on even harmonics for a low-frequency sinusoidal waveform using a high-power acoustic extinguisher. * R^2^ = 1 (order no 16); R^2^ = 0.9469 (order no 14); R^2^ = 0.9749 (order no 12); R^2^ = 0.905 (order no 10).

**Figure 8:**
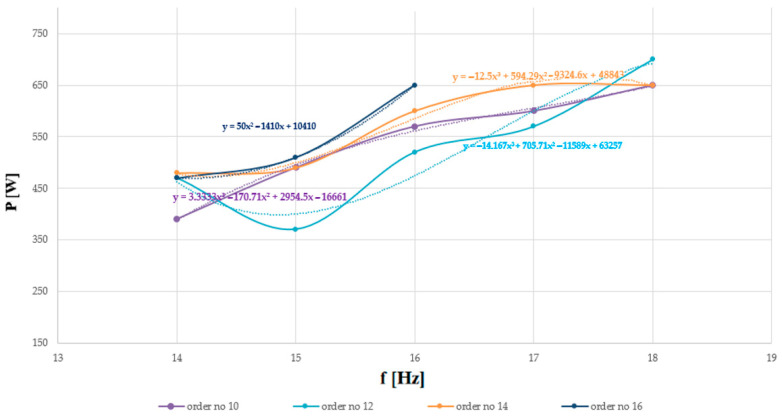
P [W] versus f [Hz] depending on even harmonics for a low-frequency sinusoidal waveform using a high-power acoustic extinguisher. * R^2^ = 1 (order no 16); R^2^ = 0.9853 (order no 14); R^2^ = 0.9328 (order no 12); R^2^ = 0.9966 (order no 10).

To facilitate the interpretation of the data, the article also includes Figure 7 and Figure 8, which present diagrams obtained for all extinguishing attempts, depending on the fundamental frequency and the harmonic order analyzed. This means that Figure 7 and Figure 8 present the SPL and P diagrams obtained not only for the boundary harmonic orders, but also for all harmonic orders analyzed in this study (i.e., from the 10th to the 16th). In Table 1 below, the results obtained for waveforms containing harmonics up to the tenth order (inclusive) are provided. As the frequency of the sound wave increased, an increase in the SPL was observed.

For a fundamental frequency of 14 Hz and higher harmonics up to the tenth order (inclusive), the sound pressure at the site where the flames were extinguished was equal to 125.6 dB. An increase in frequency of 1 Hz resulted in an increase of 0.1 dB in the SPL (it was equal to 125.7 dB). At a fundamental frequency of 16 Hz, a sound pressure of 126.4 dB was recorded (an increase of 0.7 dB over the previous pressure value). Interesting in this context are the results obtained for the operating frequency for which the extinguisher was designed, i.e., 17 Hz. For this fundamental frequency, the measured SPL was equal to 129.2 dB (an increase of 2.8 dB over the previous value due to the favorable mechanical loading of the sound-source diaphragm). In turn, when moving away from the optimal frequency for which the extinguisher was designed—18 Hz—the measured SPL at the site where the flames were extinguished decreased slightly and was equal to 127.4 dB.

Table 2 shows the results, which refer to the power that had to be delivered to the acoustic extinguisher to observe the phenomenon of completely extinguishing the flames, depending on the fundamental frequency and harmonic order. As in the case of sound pressure, as the frequency increased, a gradual increase in the power needed to be delivered to the extinguisher to extinguish the flames was observed. The highest SPL at the site where the flames were extinguished was recorded at 17 Hz (the measured power was then 600 W). In practice, the SPL and the electrical power delivered to the fire extinguisher have a logarithmic relationship.

At a fundamental frequency of 14 Hz, the lowest measured power that had to be delivered to the extinguisher to extinguish the flames was equal to 390 W. However, the problem turned out to be the large amplitude of oscillation of the sound source’s diaphragm (design limitations), which limits the use of low-frequency acoustic waves. Increasing the frequency by 1 Hz resulted in an increase in the power level by 100 W compared to the previous measurement. At a fundamental frequency of 16 Hz, a power level of 570 W was recorded (an increase of 80 W over the previous value). Increasing the frequency resulted in an increase in the measured power that had to be delivered to the extinguisher to extinguish the flames. For the 17 Hz frequency, it was equal to 600 W (the oscillation amplitude of the speaker’s diaphragm was small then), while for 18 Hz it was equal to 650 W, which corresponds to the power increases of 30 and 50 W, respectively, compared to the previous measurements.

Below, in Table 3, the results obtained for distorted waveforms containing harmonics up to the sixteenth order (inclusive) are presented. As before, there was an increase in SPL with increasing frequency of the acoustic waveform, but for this order of harmonics (due to design reasons), the measurements were carried out in a limited range.

For a fundamental wave frequency of 14 Hz and higher harmonics up to the sixteenth order (inclusive), the sound pressure at the site where the flames were extinguished was equal to 125.0 dB. Increasing the frequency by 1 Hz resulted in an unexpected slight decrease of 0.2 dB in the SPL (it was equal to 124.8 dB), which can be explained by a short-term change in atmospheric conditions. At a fundamental frequency of 16 Hz, a sound pressure of 126.3 dB was recorded (a 1.5 dB increase over the previous value). It should be noted that at 650 W, due to an excessive temperature increase, the thermal protection installed in the power amplifier tripped, so no further attempts were made for fear of damaging the equipment.

Table 4 presents the results, which refer to the power that had to be delivered to the acoustic extinguisher to observe the phenomenon of complete flame extinguishment, depending on the fundamental frequency and harmonic order. As in the case of sound pressure, as the frequency increased, a gradual increase in the power that had to be delivered to the extinguisher to effectively extinguish the flames was observed.

Analogous to the previous fire-extinguishing attempts, the lowest power was recorded for the lowest fundamental frequency analyzed. At a fundamental frequency of 14 Hz, the lowest measured power that needed to be delivered to the extinguisher to extinguish the flames was equal to 470 W. However, as previously mentioned, the use of too-low frequencies is associated with limitations due to the significant amplitude of oscillation of the sound source’s diaphragm. Increasing the frequency by 1 Hz resulted in a 40 W increase in power compared to the previous measurement. At a fundamental frequency of 16 Hz, a power of 650 W was recorded (an increase of 140 W over the previous measurement). At 650 W, the thermal protection tripped, and therefore, no further attempts were made due to the possibility of damage to the equipment. However, it was possible to see from the experiments that increasing the frequency resulted in an increase in the power that had to be delivered to the extinguisher to effectively extinguish the flames.

Comparing the results obtained for the boundary distorted sinusoidal waveforms containing higher harmonics in addition to the fundamental component, that is, up to the tenth and sixteenth order (inclusive), for the smallest of the analyzed frequencies, i.e., 14 Hz, a difference of 0.6 dB was obtained in the measured SPL at the site where the flames were extinguished. For sine waves with a fundamental frequency of 15 Hz, the recorded difference was equal to 0.9 dB, while for the waves with the highest fundamental frequency for which results were obtained, both in the case of extinguishing the flames with waves containing harmonics up to the tenth and sixteenth orders (inclusive), i.e., 16 Hz, the measured difference was equal to 0.1 dB. For higher harmonics up to the sixteenth order, no SPL results were obtained for 17 and 18 Hz because the thermal protection in the power amplifier tripped. For higher harmonics up to the tenth order, thermal protection tripped for waveforms above 18 Hz. A comparative analysis of the power that had to be delivered to the extinguisher in order to effectively suppress the flames showed that, for the lowest of the analyzed fundamental frequencies (14 Hz), boundary-distorted sinusoidal waveforms containing higher harmonics in addition to the fundamental component resulted—depending on the harmonic order—in a power difference of 80 W at an SPL difference of 0.6 dB. For a fundamental frequency of 15 Hz, the recorded power difference was 20 W (with an SPL difference of 0.9 dB), whereas for waveforms with a fundamental frequency of 16 Hz, the power difference reached 80 W, with the corresponding SPL difference being 0.1 dB.

During earlier experimental attempts, for the smallest of the analyzed fundamental frequencies, that is, 14 Hz and harmonics of the second order, the minimum power recorded was less than 100 W; for 14 Hz and harmonics of the sixth order it was more than twice as much, i.e., 240 W; and for the same frequency and harmonics of the eighth order it was equal to 340 W, while for 14 Hz and harmonics of the tenth order the necessary minimum power was recorded at 390 W (a difference of about 300 W compared to a waveform of the same frequency but harmonics of the second order) [89]. The results obtained indicate that, with an increase in the fundamental frequency, for a given harmonic order, there is a need to gradually increase the power supplied to the sound source in order to observe the phenomenon of complete flame extinction, accompanied by a recorded increase in the sound pressure level. For waveforms containing second-order harmonics, depending on the fundamental frequency (varied in the range from 14 to 20 Hz with a step of 1 Hz), the power ranged from 85 to 580 W (the sound pressure level at which flame extinction was observed ranged from 120.7 to 126.9 dB). For waveforms containing harmonics up to the fourth order (inclusive), the power varied from 210 to 450 W. The recorded sound pressure level at the flame-extinction location for the extreme frequencies, i.e., 15 and 18 Hz, then ranged from 124.6 to 126.3 dB. For waveforms containing harmonics up to the sixth order, a power level of 240–590 W was required (corresponding to frequencies of 14 and 19 Hz, respectively). The measured sound pressure level then ranged from 124.2 to 126.7 dB. In turn, for waveforms containing harmonics up to the eighth order, depending on the fundamental frequency, the power varied from 270 to 900 W (the recorded sound pressure level at which complete flame extinction was observed ranged from 126.1 to 127.4 dB).

These experiments carried out also allow for some considerations. Assume that a candle burns about 3 to 8 g of wax per hour and generates a thermal power of 38 to 100 W, that is, an average of about 70 W, a power of about 100 to 400 W at 14 Hz and about 600 to 900 W at 20 Hz, depending on the fundamental frequency, is required for a candle heating power of less than 100 W, based on the acoustic data presented in [89]. In practice, a much higher power sound source is used to extinguish a 100 W candle. This means that a large amount of electrical power is required to extinguish a candle with a small flame power, which is determined by the physical capabilities and extinguishing capacity of the acoustic waves. The issue of selecting a sound source (e.g., other than a woofer) may prove crucial in the future. However, these aspects require further research and are beyond the scope of this article.

As demonstrated in this study, a similar analogy can also be observed for other fundamental frequencies and higher harmonic orders. With each successive increase in the fundamental frequency by 1 Hz, for a given harmonic order, a need was observed to gradually increase the power supplied to the sound source in order to extinguish the flames, accompanied by a corresponding increase in the recorded sound pressure level. For waveforms containing harmonics of the lowest order analyzed in this study, i.e., the tenth order, depending on the fundamental frequency, the power varied in the range from 390 to 650 W (the sound pressure level at which complete flame extinction was observed then ranged from 125.6 to 127.4 dB). For distorted waveforms containing harmonics up to the 12th order (inclusive), the power varied from 470 to 700 W. The recorded sound pressure level at the flame-extinction location for the extreme frequencies, i.e., 14 and 18 Hz, then ranged from 125.6 to 127.3 dB. For waveforms containing harmonics up to the 14th order, a power level of 480–650 W was required (corresponding to frequencies of 14 and 18 Hz, respectively). The measured sound pressure level then ranged from 125.2 to 126.3 dB. In turn, for waveforms containing harmonics of the highest order analyzed in this study, i.e., the sixteenth order (inclusive), depending on the fundamental frequency, the power varied from 470 to 650 W (the recorded sound pressure level at which complete flame extinction was observed then ranged from 125.0 to 126.3 dB).

On the basis of an analysis of all the results obtained, it can be concluded that increasing the order of harmonics affects the gradual increase in the power that must be delivered to the extinguisher to observe the phenomenon of complete extinguishing of the flames. The sound pressure for distorted waveforms containing harmonics up to the tenth and sixteenth orders (inclusive), at which complete extinguishment of the flames was recorded, oscillates in the range of about 125–129 dB. The experiments demonstrated that harmonics within the analyzed frequency range affect flame extinguishing and that the fundamental frequency has a significant influence on the extinguishing process.

The diagrams were plotted as functions of the fundamental frequency and the maximum order of the even harmonics present in the signal, namely the tenth, twelfth, fourteenth, and sixteenth orders. To estimate (predict) the power that is needed for the fire extinguisher to observe the phenomenon of complete extinguishing of the flames, as well as the SPL at the site where their extinguishment was recorded, one can use the empirical formulas by applying the classical regression function (in this case, third-order polynomials). These formulas allow for the prediction of future SPL and P values based on known values obtained from experimental attempts, which can serve as reference points, while accounting for the non-linear nature of the observed phenomena. The use of regression techniques allows for modeling non-linear relationships between variables. As noted above, when very low-frequency waves are applied, a significant vibration amplitude of the speaker diaphragm is observed, which may limit their practical use. Since the measurements were obtained during the same experiments, it can be observed that the curves representing the minimum power level required to be delivered to the sound source and the sound pressure measured at the location where flame extinguishment occurred exhibit similar shapes.

Analysis of the extinguishing attempts shows that the SPL at which flame extinguishment was recorded ranged from 124.7 to 129.2 dB, depending on the frequency and order of harmonics (inclusive). As the frequency increased, an increase in the SPL required to extinguish the flames was observed, regardless of the order of the higher harmonics. Any discrepancies may be attributed to temporary variations in the conditions prevailing during the experimental trials (e.g., wind gusts). In practice, a similar trend was observed previously when analyzing the effect of harmonics on flame extinguishment.

Diagrams of the power that had to be delivered to the acoustic extinguisher to observe the phenomenon of complete extinguishment of the flames, depending on the fundamental frequency and harmonic order, are presented in Figure 8.

Based on the diagrams in Figure 8, it can be seen that, as the frequency increases, an increase in the power needed for the acoustic extinguisher is observed to completely extinguish the flames. This trend was observed regardless of the order of the higher harmonics and, from a statistical standpoint, it represents a consistently increasing trend. Increasing the order of harmonics to the sixteenth (inclusive) resulted in the need to increase the power required to extinguish the flames at the same frequency. For example, for the smallest of the analyzed frequencies, i.e., 14 Hz and harmonics up to the tenth order (inclusive), the power that had to be delivered to the extinguisher to extinguish the flames was equal to 390 W, while for harmonics up to the sixteenth order, it was equal to 470 W (an increase of 80 W). In turn, for the largest of the joint fundamental frequencies analyzed in this experiment, i.e., 16 Hz and harmonics up to the tenth order (inclusive), the power that had to be delivered to the extinguisher to extinguish the flames was equal to 570 W, while for harmonics up to the sixteenth order (inclusive), it was equal to 650 W (also an increase of 80 W). Analysis of the data obtained for non-boundary harmonic orders shows that, for the fundamental waveform and harmonics up to the twelfth order at a frequency of 18 Hz, the power required to be delivered to the extinguisher to extinguish the flames was equal to 700 W. This was the highest measured power value for this harmonic order during this experimental series, recorded before the power amplifier’s thermal protection was triggered. In practice, the extinguisher allowed quick and effective firefighting action. Based on the obtained results, it can be concluded that increasing both the fundamental frequency and the harmonic order leads to a higher power requirement that must be delivered to the extinguisher to successfully extinguish the flames. The results experimentally confirm the validity of the hypothesis that the process of extinguishing the flames is affected by the presence of higher-order harmonics in addition to the fundamental frequency.

**Figure 9:**
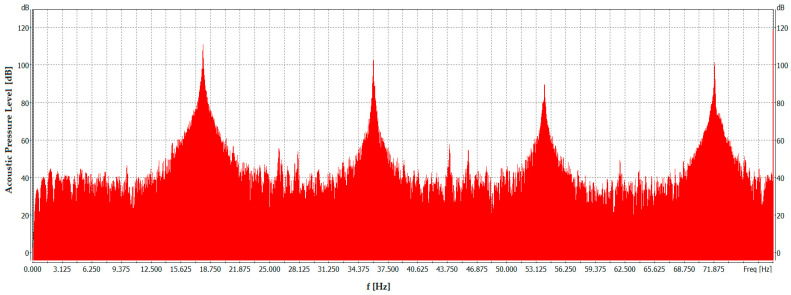
Fast Fourier transform graph for a fundamental frequency of 18 Hz and harmonics up to the 10th order.

Below, in Figure 9, for illustrative purposes, is an FFT graph showing the dependence of the sound pressure as a function of frequency at the point where the flames were extinguished. On this basis, we can see which frequencies exist in vibration over a certain period of time (for the samples, it can be determined how many frequencies are present). The frequency was presented on the X axis, while the amplitude of the SPL was on the Y axis. Interpretation of the results obtained confirmed the presence of harmonics that affect flame extinguishing. Spectrum measurements, as in the work [89], were made according to the ANSI S.12.2-2008 standard. In practice, the FFT bandwidth was equal to 78.125 Hz, the number of FFT lines was equal to 1601, and the FFT sampling frequency was equal to 51200 Hz.

**Figure 10:**
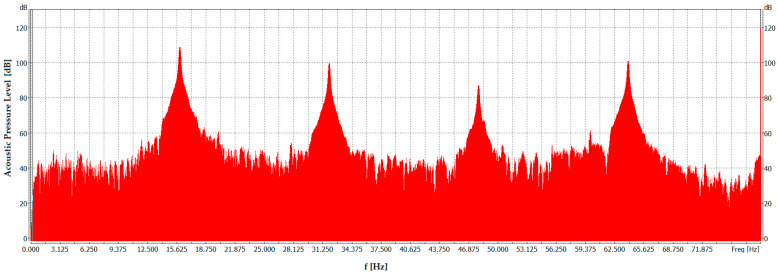
Fast Fourier transform graph for a fundamental frequency of 16 Hz and harmonics up to the order 16.

The presented spectrum of acoustic pressure level as a function of frequency was obtained for the highest fundamental frequency, i.e., 18 Hz, at which measurements were possible for a signal containing harmonics up to the tenth order. Analysis of this spectrum reveals clear peaks at multiples of the fundamental frequency (18 Hz), indicating the presence of dominant harmonics in the resulting distorted waveform used for flame extinguishment. Peaks with high SPLs are shown in Figure 9. For comparison, Figure 10 presents an analogous graph obtained at the highest fundamental frequency for which measurements were successfully conducted with harmonics up to the sixteenth order, i.e., 16 Hz.

As shown in Figure 10, the spectrum is dominated by higher harmonics compared with that in Figure 9. In this case, the signal spectrum exhibits distinct peaks at frequencies that are multiples of 16 Hz (fundamental frequency), that is, 32 Hz, 48 Hz, and 64 Hz (the peaks exceed the 100 dB level). The signal spectrum is influenced by additional harmonics of the twelfth, fourteenth, and sixteenth orders, which were not present in the signal spectrum in Figure 9. It translates into amplitude changes for specific frequencies.

**Figure 11:**
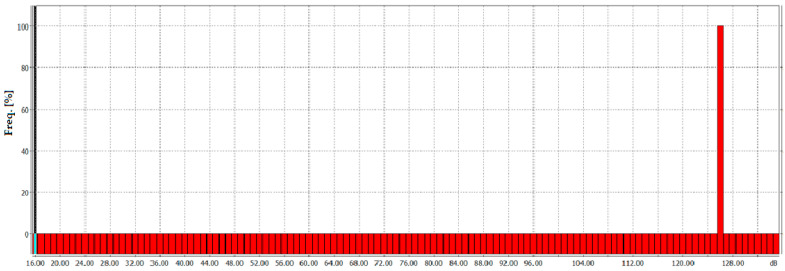
Histogram showing the frequency distribution for equivalent sound level values.

In addition to spectral plots, histograms are also presented. Their construction makes it possible to visualize dominant values (trends and deviations from the norm) as well as the range of their variability. For illustrative purposes, Figure 11 provides an example histogram showing the frequency distribution of the analyzed variable. The SPL measured at the site of the extinguished flames was applied (this means equivalent sound level values using the Z-correction frequency characteristic). The resulting percentage of occurrences for each range of values is posted on the Y axis.

On this basis, it is possible to present the frequency distribution and read the value of the measured SPL (in this case, it was equal to 126.9 dB). This means that the recorded occurrence rate of the SPL for the analyzed signal was equal to 100 percent, which confirms the correctness of the implementation of the experiment.

**Figure 12:**
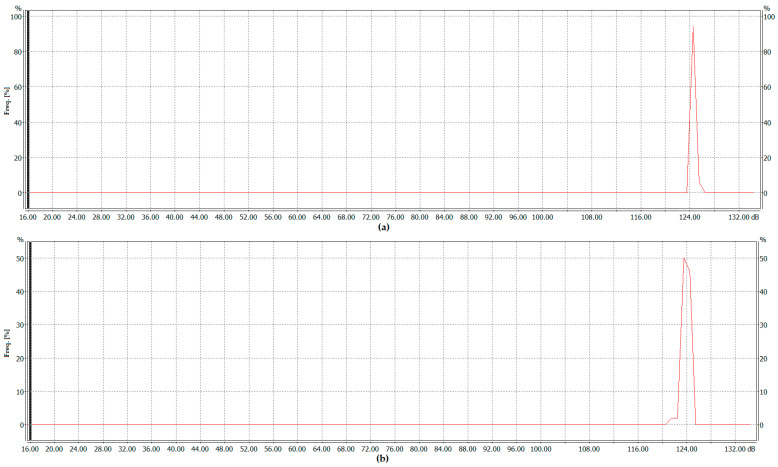
Histograms showing the frequency distribution for equivalent sound level values.

While Figure 9 and Figure 10 present results obtained at the highest signal frequencies for which measurements were successfully conducted (i.e., 18 Hz for harmonics up to the tenth order and 16 Hz for harmonics up to the sixteenth order, inclusive), corresponding to the highest power levels required to extinguish the flames, Figure 12 presents histograms showing the frequency distribution of equivalent sound level values measured at the lowest frequency for which measurements were successfully conducted. In both cases, this frequency was 14 Hz, at which the power required to be delivered to the acoustic extinguisher was the lowest. The histogram for a signal containing harmonics up to the tenth order (inclusive) is shown in Figure 12a, and the histogram for a signal containing harmonics up to the sixteenth order (inclusive) is presented in Figure 12b.

An analysis of the frequency distribution as a function of the SPL shows that the maximum frequency value reaches 100%, indicating stable experimental conditions. This observation is further supported by the summaries presented in Table 5 and Table 6. In practice, nearly all results are concentrated within a narrow range at high SPLs. Any observed anomalies or fluctuations may be attributed to short-term changes in conditions during the experimental attempts. The equivalent sound level values obtained using the Z-correction characteristic and boundary levels exceeded by the temporary noise level during no more than n% of the observation period achieved for this frequency are presented in Table 5 and Table 6.

The obtained equivalent sound level oscillates around a median value of 125.5 dB, indicating that the recorded sound pressure was above this level for half of the observation period and below it for the remaining half. In practice, during the experiment, the level of the sound pressure threshold of 125.9 dB was exceeded by no more than 10% of the observation period, the level of 125.5 dB was exceeded by no more than 50% of the observation period, and the level of the sound pressure threshold of 125 dB was exceeded by no more than 95% of the observation period (meaning that it was greater than 125 dB 95% of the time). The difference between the L10 and L95 values is only 0.9 dB, indicating that the SPL remained almost constant over time during the firefighting attempts. An analogous list containing the equivalent sound level and threshold levels exceeded for at most n% of the observation period, for a signal containing harmonic components up to the sixteenth order (inclusive), is given in Table 6.

As in the previous case, the SPLs measured during the experimental attempts were high. The boundary SPL of 125.7 dB was exceeded for no more than 10% of the observation period, 124.9 dB for no more than 50% of the period, and 124.0 dB for no more than 95% of the period, indicating that the sound pressure exceeded 124 dB during 95% of the observation time. In this case, the difference between the L10 and L95 values was equal to 1.7 dB, confirming that the SPL during the experimental measurements was relatively constant and did not fluctuate significantly, although it was slightly less stable than in the previous attempts. In practice, analogous graphs and tables were obtained for all other analyzed frequencies and harmonic orders. However, due to the large volume of data and the limited length of the manuscript, they were omitted from this paper. Only the most relevant data illustrating the observed relationships between the parameters of low-frequency distorted waveforms and flame extinguishment were included. The results obtained allow one to complement the state-of-the-art in the use of low-frequency distorted waveforms containing higher harmonics for the purpose of extinguishing flames with acoustic waves. In this context, the results experimentally confirm that the flame extinguishing process is influenced not only by the fundamental frequency but also by the presence of higher-order harmonics within the analyzed technical parameters of the acoustic wave.

## 4. Limitations, Future Challenges, and Potential Applications

In practice, firefighting mainly uses chemicals selected according to the class of fire. Although techniques using foam, powder, snow, water, and water-mist extinguishers are effective, they have an adverse effect on the objects extinguished or the environment, and as a consequence, some solutions are being withdrawn from use (an example in many countries is the halon extinguisher). In addition, in the case of classical fire protection solutions, their short-lasting extinguishing effect, difficult transport to the site of the firefighting action, and the fact that the replenishment of the tanks is handled only by specialized services and that this activity is time-consuming, are problematic. This is important because in the event of a fire, any time delay leads to an increase in immaterial and material losses. Therefore, it is necessary to look for more sophisticated ways to fight fires.

Since 2022, acoustic wave-based flame extinguishing technology has been actively investigated and further developed, demonstrating significant potential for practical applications. In practice, the shape, type of modulation, frequency, and harmonic order influence the flame extinguishing process [16,17,19,20,23,24,25,31,32,35,42,44,45,89,94]. Therefore, to enable broader and more reliable implementation, further systematic research is required to thoroughly characterize the extinguishing capability of low-frequency acoustic waves generated by high-power sound sources as a function of the fire source and fire class. Such studies will allow the operational limits of this technique to be clearly defined and its future applications to be explored. For effective operation, the acoustic source should be positioned within the acoustic field. The acoustic method is characterized by lower operating and extinguishing costs compared with powder-, foam-, and gas-based systems, as well as by its non-invasive nature, resulting from the fact that acoustic waves are non-chemical and invisible to humans, causing no fog-like effects, pollution, or collateral damage, which are typically associated with firefighting foams or water. In addition, acoustic extinguishers are not subject to periodic tank pressure testing, as required for conventional extinguishers (e.g., every 10 years), and they offer virtually unlimited operating time, provided that mains or battery power is available. Another unquestionable advantage is the ability to tailor the parameters of the extinguishing wave to a specific fire source. When addressing different flame sources, a frequency-sweeping technique may be applied [19]. Consequently, the acoustic method appears to be a cost-effective alternative to traditional firefighting approaches, offering versatility, non-consumable operation, and unrestricted duration. In practice, acoustic waves enable effective flame extinguishment.

As demonstrated in the article, the electrical power required to achieve an extinguishing effect can be increased by the use of higher-frequency waves. Lower-frequency acoustic waves are considered more effective because they induce higher-amplitude flame vibrations, resulting in superior extinguishing efficiency. In testing, the highest efficiency was demonstrated by the lowest frequency examined (i.e., 14 Hz), with the least electrical power required. Therefore, it is logical for acoustic extinguishers to be designed to operate at the lowest frequencies possible. However, significantly longer waveguides are required to generate waves at only a few Hertz, which presents the practical implementation challenges mentioned earlier. A threshold frequency of approximately 20 Hz was observed, beyond which the extinguishing effect became difficult to achieve [20]. On the other hand, distinct advantages are offered by the utilization of higher-frequency waves. As frequency is increased, the acoustic beam becomes more concentrated and directional, whereby the device’s effective range is directly improved. Additionally, a more compact extinguisher design is allowed for by higher frequencies—a critical factor for mobile applications. Nevertheless, it is confirmed by experimental research that higher electrical power consumption is necessitated by these higher frequencies.

In the future, this method may find application in locations where access to conventional fire protection measures is limited, where the amount of extinguishing agent is severely restricted, or where flames originate from materials that are difficult to suppress using currently available methods. This is particularly relevant given the ongoing global research into new materials (e.g., [95,96,97,98,99,100,101,102,103,104]) as well as innovative solutions for fire protection applications (e.g., [105,106,107,108,109,110,111,112]). In practice, the acoustic method has proven effective in extinguishing flames originating from liquids and gases, despite the combustion process being influenced by various external factors, and it has also demonstrated high effectiveness in suppressing flames generated by organic materials. A significant advantage of this technique is its potential applicability in both indoor and outdoor environments, although this requires further extensive investigation. Acoustic extinguishers may be permanently installed during the construction stage of facilities, vehicles, or installation passages, or alternatively deployed as mobile (portable) devices. Beyond industrial facilities, such as production lines, server rooms, and rack stations, this technology may also find applications in the space, aviation, land transportation (e.g., trains), and maritime sectors (e.g., ships), where favorable waveguide lengths are typically not a limiting factor. Another potential application of acoustic waves is the extinguishing of fires in transformer stations, where fires are often particularly difficult to suppress. However, the effectiveness of this method in such scenarios has not yet been verified and requires dedicated experimental studies. In practice, fires in transformer stations are challenging to extinguish and can result in extensive damage, the cost of which is often disproportionately higher than the estimated cost of implementing an acoustic extinguishing system. Therefore, similar experiments are planned for future research. If positive results are obtained, the relatively low system cost could ensure the cost-effectiveness of deploying this technique across a wide range of applications. The goal is to develop a modern autonomous acoustic fire extinguisher that incorporates artificial intelligence, which is planned to be directly integrated with the fire protection system and capable of making decisions autonomously. Such a system could detect fire or smoke by identifying anomalies in pixel color and, upon positive detection of flames, automatically notify fire protection services while simultaneously activating the flame-extinguishing process. The permanent integration of smart acoustic extinguishers or integrated acoustic extinguishing systems into commercial units is also a potential future application.

## 5. Conclusions

Acoustic technology represents an innovative and environmentally friendly approach to firefighting. At its current stage of development, it can be applied to suppress fires either as a primary method or as a complementary technique supporting other extinguishing processes, such as water mist systems. However, further research is required to assess the impact of low-frequency acoustic waves on living organisms (fauna) and humans—particularly regarding the human auditory pain threshold—as well as on building structures and interior elements, including walls and windows. In practice, additional studies are needed to evaluate the mechanical strength and durability of materials subjected to prolonged exposure to low-frequency waves, even though such waves are effective in flame suppression, do not contaminate extinguished surfaces, and may induce vibrations. The identified research areas require interdisciplinary investigations spanning multiple scientific fields, including medicine. Notably, human presence during firefighting operations is not necessarily required when acoustic extinguishers are used as components of automated firefighting systems integrated with intelligent (smart) sensors—particularly for outdoor applications—or with conventional temperature and smoke detectors for indoor facilities. In practice, the selection of a specific firefighting strategy depends on the intended application environment. Equipping an acoustic extinguisher with an intelligent smart module, including advanced sensors and artificial vision systems, enables automatic activation immediately upon fire detection (the firefighting system can be autonomously triggered to initiate flame suppression). In the event of positive detection, extinguishing can be activated, and relevant emergency services may be notified in real time. In the era of rapid advances in artificial intelligence, continued improvements in computer vision performance can be expected. In this sense, the smart acoustic technique serves not only as an extinguishing mechanism but also as an integral component of the fire detection process. This is achieved through the use of intelligent sensing and data analysis, whereby both technologies are able to complement each other effectively. Such an integrated solution enhances system responsiveness, shortens reaction time, and enables fully autonomous operation, which is particularly important when this technique is deployed in remote and hard-to-access locations. One limitation of acoustic extinguishers is their physical size; however, ongoing advances in the miniaturization of electronic components and materials science research may facilitate the development of smaller, more efficient, and intelligent high- and very-high-power acoustic extinguishers capable of generating SPLs sufficient to enable rapid and effective firefighting at considerable distances from the flame source.

The studies presented in this work experimentally confirm that flame extinguishing is influenced not only by frequency but also by the harmonic order. Effective flame suppression was demonstrated using modified acoustic distorted waveforms containing higher even harmonics in addition to the fundamental frequency. For this purpose, a high-power acoustic extinguisher and waveforms comprising harmonics up to the sixteenth order (inclusive) were employed. From a physical perspective, acoustic waves interact with flames primarily through local pressure oscillations, induced airflow, and turbulence generated within the flame zone. In practice, the level of turbulence depends not only on the frequency but also—as demonstrated in this article—on the harmonic order. Modifying the waveforms to include higher harmonics results in flame destabilization through temporal and spatial variations in the pressure distribution of the acoustic field, which in turn leads to extinguishing effects once the critical frequency is reached.

The fundamental frequency constitutes a key parameter, as it largely determines the scale of air particle motion and governs the interaction with the extinguished flame. The application of higher even harmonics affects the compression and rarefaction cycles (local increases and decreases in air molecule density), thereby influencing the mixing process at the flame boundary and, consequently, disrupting the combustion process at different power levels.

In the course of the conducted experiments using low-frequency distorted acoustic waves containing higher even harmonics, it was observed that these harmonics affect the threshold of electrical power that must be supplied to the sound source in order to observe the phenomenon of complete flame extinction (a shift in the threshold level). Turbulence induced by the generation of waves containing higher harmonics plays an important role in this process. Under these conditions, the recorded level of induced turbulence—necessary to disrupt the flame front—proved sufficient to enable rapid and effective extinguishment. The SPL at which flames were extinguished ranged from 124.7 to 129.2 dB for distorted waveforms containing harmonics up to the tenth and sixteenth orders (inclusive), respectively. The measured acoustic power ranged from approximately 400 W to 650–700 W for the highest analyzed frequency, for which the influence of harmonics on the flame extinguishing process was investigated. In practice, increasing both the fundamental frequency and the harmonic order resulted in a higher power requirement to achieve complete flame extinguishment. Consequently, the effectiveness of low-frequency acoustic waves is associated with their stronger interaction with air and fuel flow (lower frequencies correspond to longer wavelengths), more efficient disruption of the oxygen supply at the flame site due to local pressure variations, and favorable propagation characteristics, as such waves are less susceptible to attenuation. Nevertheless, the use of low-frequency acoustic waves also entails certain limitations, which are discussed in this article. Given that the available oxygen concentration in enclosed spaces is lower than in open environments, this technique is considered particularly promising for extinguishing flames in enclosed facilities.

## Figures and Tables

**Table 1:** SPL values as a function of frequency (f) and even harmonics (up to the order of 10) for a low-frequency sinusoidal waveform using a high-power acoustic extinguisher.

f [Hz]	SPL [dB]
14	125.6
15	125.7
16	126.4
17	129.2
18	127.4

**Table 2:** Minimum electrical power required to extinguish flames as a function of frequency (f) and even harmonics (up to the order of 10) for a low-frequency sinusoidal waveform using a high-power acoustic extinguisher.

f [Hz]	P [W]
14	390
15	490
16	570
17	600
18	650

**Table 3:** SPL values as a function of frequency (f) and even harmonics (up to the order of 16) for a low-frequency sinusoidal waveform using a high-power acoustic extinguisher.

f [Hz]	SPL [dB]
14	125.0
15	124.8
16	126.3

**Table 4:** Minimum electrical power required to extinguish flames as a function of frequency (f) and even harmonics (up to the order of 16) for a low-frequency sinusoidal waveform using a high-power acoustic extinguisher.

f [Hz]	P [W]
14	470
15	510
16	650

**Table 5:** Equivalent sound level Leq [dB] and threshold levels (Ln%) exceeded for at most n% of the observation period for a signal containing harmonics up to the order 10.

Statistical Level Ln [%]	Leq [dB]
L10	125.9
L20	125.8
L30	125.7
L40	125.6
L50	125.5
L60	125.4
L70	125.3
L80	125.2
L90	125.1
L95	125.0

**Table 6:** Equivalent sound level Leq [dB] and threshold levels (Ln%) exceeded for at most n% of the observation period for a signal containing harmonics up to the order 16.

Statistical Level Ln [%]	Leq [dB]
L10	125.7
L20	125.5
L30	125.3
L40	125.1
L50	124.9
L60	124.7
L70	124.5
L80	124.3
L90	124.1
L95	124.0

## Data Availability

Data are contained within the article.
